# A novel workflow for multi‐modal imaging of musculoskeletal tissues

**DOI:** 10.1111/joa.14202

**Published:** 2025-01-17

**Authors:** Anya König, Brenton L. Cavanagh, Isabel Amado, Amit Kalra, Bohnejie A. Ogon, Paige V. Hinton, Oran D. Kennedy

**Affiliations:** ^1^ Department of Anatomy and Regenerative Medicine Tissue Engineering Research Group (TERG) Royal College of Surgeons Ireland (RCSI) University of Medicine and Health Sciences Dublin Ireland; ^2^ Cellular and Molecular Imaging Core Royal College of Surgeons Ireland (RCSI) University of Medicine and Health Sciences Dublin Ireland; ^3^ Trinity Center for Biomedical Engineering Trinity College Dublin Dublin Ireland; ^4^ Advanced Materials and BioEngineering Research (AMBER) Centre Dublin Ireland

**Keywords:** bone, cartilage, imaging, MicroCT, microscopy, musculoskeletal, PACT, SHG

## Abstract

According to the World Health Organization (WHO) musculoskeletal conditions are a leading contributor to disability worldwide. This fact is often somewhat overlooked, since musculoskeletal conditions are less likely to be associated with mortality. Nonetheless, treatments, therapies and management of these conditions are extremely costly to national healthcare systems. As with all systemic conditions, biomedical imaging of relevant tissues plays a major role in understanding the fundamental biological processes involved in musculoskeletal health. However, the skeletal system with its relatively large proportion of dense, opaque (often mineralised) tissues can often be more challenging to image, and recently important advances have been made in imaging these complex musculoskeletal tissues. Thus, we here describe a novel workflow in which recent advanced imaging techniques have been modified and optimised for use in musculoskeletal tissues (specifically bone and cartilage). This will allow for investigations, of different phases of these tissues, at new and higher resolutions. Furthermore, the process has been designed to fit with the existing and standard processes which are typically used with these samples (i.e. μCT imaging and standard histology). The additional modalities which have been included here are second harmonic generation (SHG) imaging, tissue clearing, specifically the Passive Clear Lipid‐exchanged Acrylamide‐hybridised Rigid Imaging Tissue hYdrogel (CLARITY) method known as PACT, and then imaging of these tissues with confocal, multiphoton and light‐sheet microscopy. This paper serves to introduce a combination of existing new methods and improvements in imaging of musculoskeletal tissues.

## Graphical abstract

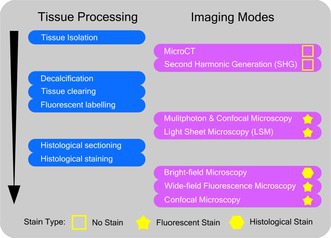

## 1 INTRODUCTION

According to the World Health Organization (WHO) and numerous systematic reviews, musculoskeletal conditions are a leading contributor to disability worldwide (Briggs et al., 2020; Liu et al., 2022; World Health Organization, 2022). Diseases of the musculoskeletal system can present at all stages of life and are not, as is often assumed, confined to the ageing process. In fact, approximately 20%–30% of people (including children) suffer from musculoskeletal pain globally (Briggs et al., 2020; Liu et al., 2022; World Health Organization, 2022) at any given time. This fact is often somewhat overlooked, since musculoskeletal conditions are less likely to be associated with mortality. Nonetheless, treatments, therapies and management of these conditions are often extensive and almost always extremely costly to national healthcare systems. In addition to the immediate impacts, long‐term symptoms of musculoskeletal injuries have been linked to depression as well as predisposition to other chronic conditions (Jin et al., 2021; Marconcin et al., 2023). The importance of musculoskeletal conditions to global populations has resulted in significant interest and investment in research to understand these diseases.

As with all systemic conditions, imaging of tissues and structures plays a major role in understanding the fundamental biological processes of those that affect musculoskeletal health. However, the skeletal system with its relatively large proportion of dense, opaque (often mineralised) extra‐cellular matrix (ECM) and relatively sparse cellularity means imaging of these tissues can often be particularly challenging. There are several well‐known, long‐standing imaging principles that work very effectively in the clinical management of musculoskeletal conditions (e.g. *x*‐ray/computed tomography (CT)/magnetic resonance (MR)‐based modalities). There are also various research/laboratory‐based techniques/technologies that are well‐established and routine for studies that focus on bone and joint diseases (e.g. micro‐computed tomography (μCT), and histology (with either biochemical or immunohistochemical staining)). Imaging modalities and techniques in all fields of research are continuously evolving and improving. However, advances tend to be made in other fields of research first, and more rapidly, due to the additional difficulty of imaging complex musculoskeletal tissues. Many research groups, including ours, have been working to address this deficit in imaging in musculoskeletal research by developing new and useful tissue preparation and imaging protocols.

One such area is the field of optical tissue clearing, which has increased our understanding of cell and molecular processes and responses in various musculoskeletal applications. For example, while traditional immunohistochemistry techniques typically require frozen or paraffin sections, optical clearing methods enable studies of tissue/cell architecture in intact whole tissues and organs. However, processing mineralised bone using these methods has proven challenging because very long clearing times are required, even with the relatively harsh treatments employed. Thus, we here describe a novel workflow in which recent advances in imaging techniques have been modified and optimised for use in musculoskeletal tissues (specifically bone and cartilage). This will allow for investigations of different phases of these tissues, at new and higher resolutions. Furthermore, the process has been designed to fit with the existing and standard processes which are typically used with these samples (i.e. μCT and histology). The additional modalities which have been included here are second harmonic generation (SHG) imaging, tissue clearing, and the Passive Clear Lipid‐exchanged Acrylamide‐hybridised Rigid Imaging Tissue hYdrogel (CLARITY) method known as PACT, and then imaging of these tissues with confocal, multiphoton and light‐sheet microscopy.

While some of these methods have been used in various ways on bone and cartilage tissues in the past—we present this as a novel workflow which allows each step to be carried out in sequence on the same sample, gleaning different and complementary information from different compartments of the tissue, at each stage of the process. This paper serves to introduce a combination of exciting new methods and improvements in imaging of musculoskeletal tissues.

## 2 METHODS AND IMAGING

### 2.1 Specimens and background

**FIGURE 1:**
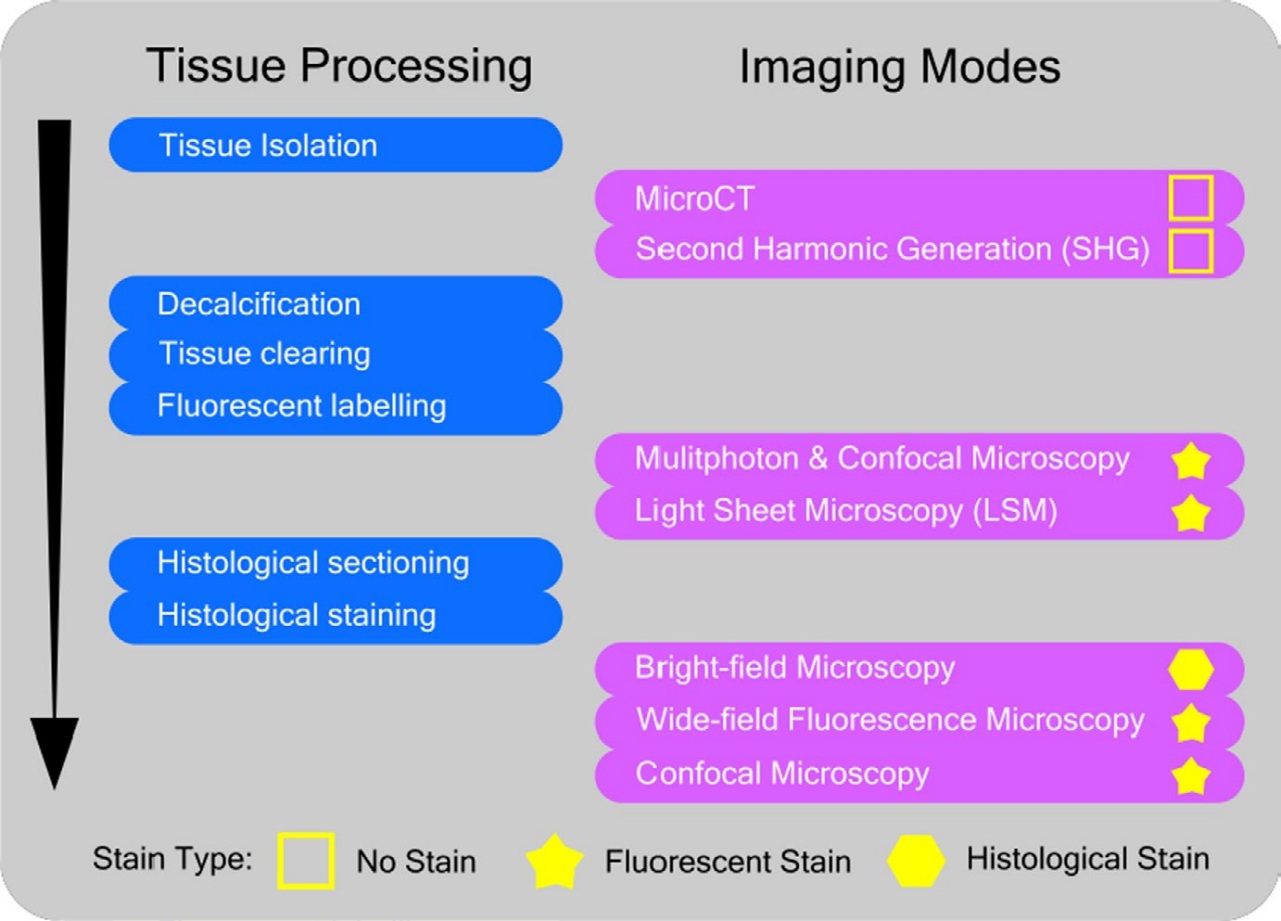
Summary of the overall procedural workflow followed in this study, showing tissue processing stages, staining procedures and imaging modes.

For all the data reported in this study, each imaging modality was used to assess either a murine flat bone (calvarium) and/or a long bone from the upper limb. The calvarium was chosen as the initial proof‐of‐principle site to test and develop these protocols, since the post‐natal development of this bone contains many of the relevant biological processes related to skeleton (bone formation, mineralisation, cell–cell communication, etc.), while still being relatively simple in shape/dimension. As a flat bone, the murine calvarium is very thin which allows for the passage of electromagnetic light waves through the tissue—which is, of course, central to many of our imaging processes. Then, once optimised, these protocols were applied to one or other of our long bone samples. This is because, while calvaria can be very useful for the initial development of these applications—most research is typically carried out on long bones for either bone or joint applications. All samples were obtained from related studies in our laboratory, under institutional license and approval, and the sourcing of these tissues was carried out in accordance with the 3Rs principles. Figure 1 provides a graphical representation of the overall procedure followed in this study.

### 2.2 Sample preparation and imaging (μCT, SHG, PACT, confocal, LSM)

#### 2.2.1 Fixation and MicroCT

**FIGURE 2:**
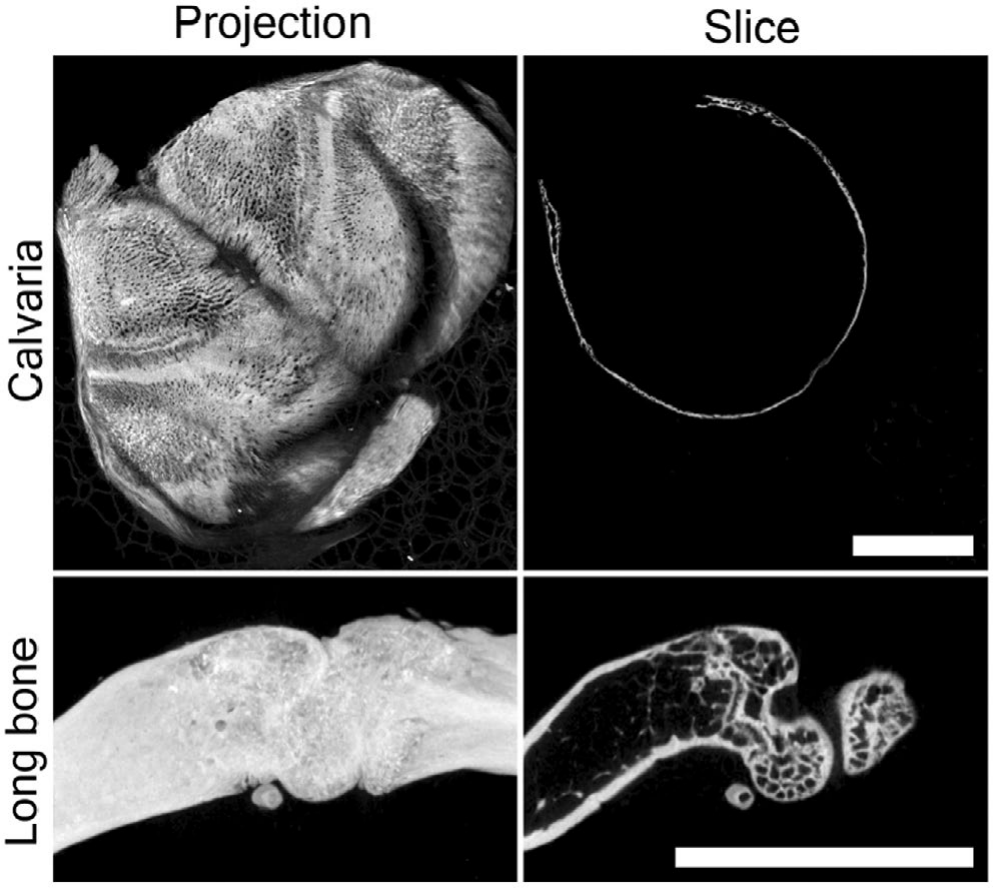
Representative microCT images of murine calvarium and long bone, presented as both a full projection (left panels) and an individual 2D slice (right panels). Scale bar = 5 mm.

All tissue samples were fixed, immediately following harvest, in 10% neutral buffered formalin (NBF) for 3 days and then rinsed and transferred to 70% ETOH until required. Next tissues were prepared for micro‐computed tomography (μCT) imaging. All μCT scanning was carried out using the Scanco μCT‐40 system and the associated software for visualisation and analysis. Samples were placed in a 15 mm sample holder and scanned at 16.4 μm resolution, 70 kVp and 114 mA *x*‐ray settings. Raw data were used to produce 2D and 3D reconstructions. Images were then processed using the manufacturer's software. This technique provides excellent structural detail of mineralised (or otherwise opaque) materials, up to a spatial resolution of approximately 10 μm, as shown in Figure 2. However, it cannot provide any ‘biological’ detail per se, such as cellular/enzymatic processes, nor discreet information on ECM proteins such as collagen distribution or orientation.

#### 2.2.2 Second harmonic generation (SHG) imaging

**FIGURE 3:**
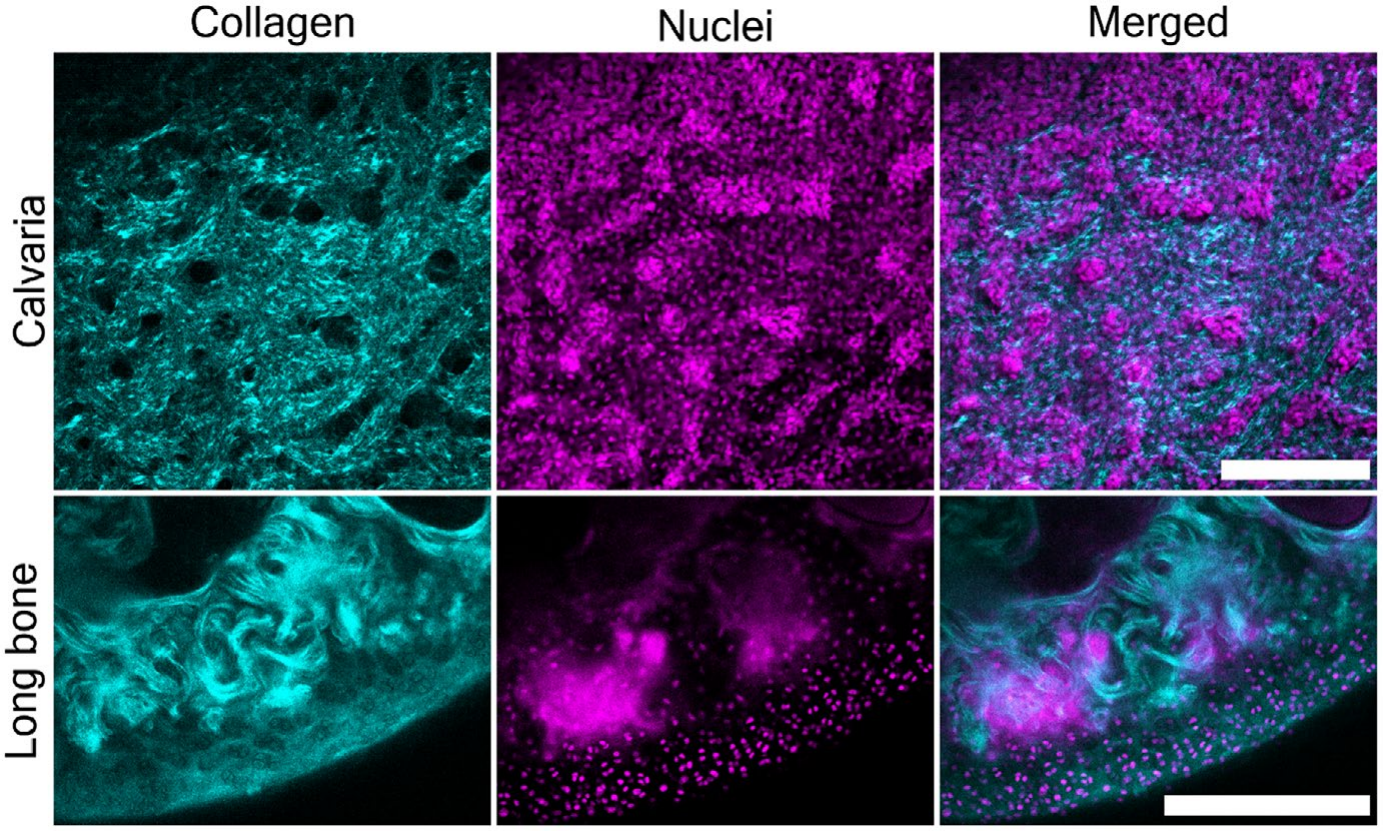
Representative images of rodent calvarium (upper panels) and long bones (lower panels) using SHG imaging (cyan), and combined with nuclear imaging (magenta, DAPI). Scale bar = 200 μm.

Thus, to address the primary component of the ECM in these tissues (i.e. collagen) we next performed second harmonic generation (SHG) imaging of our samples. SHG imaging can provide information on specific collagens in the ECM (Juan et al., 2016) in terms of content and orientation, without any requirement for specific staining or labelling. SHG imaging is a non‐linear optical imaging technique which is applied to non‐centrosymmetric structures (such as fibrillar collagen) whereby two excitatory photons simultaneously interact with the non‐centrosymmetric material, resulting in the generation and emission of a single photon with half the wavelength of the incident photons. This allows the specific visualisation of collagens I and II (Figure 3). Furthermore, even with clearing and PACT procedures, imaging was limited to a depth of approximately 100–300 μm below the surface of the tissue. Briefly, the procedures used to perform SHG imaging involved a Carl Zeiss 710 NLO confocal microscope equipped with 10 or 20× water immersion lens and a Coherent Chameleon Vision II TI: sapphire laser. For SHG, the sample was excited with unpolarised 840 nm light and the resulting 420 nm backscattered emission was collected with a non‐descanned detector and 485 nm short pass filter. Standard nuclear staining with DAPI was used to label resident cell populations. The Zen 2010 software was used for image collection.

#### 2.2.3 Decalcification and PACT

**FIGURE 4:**
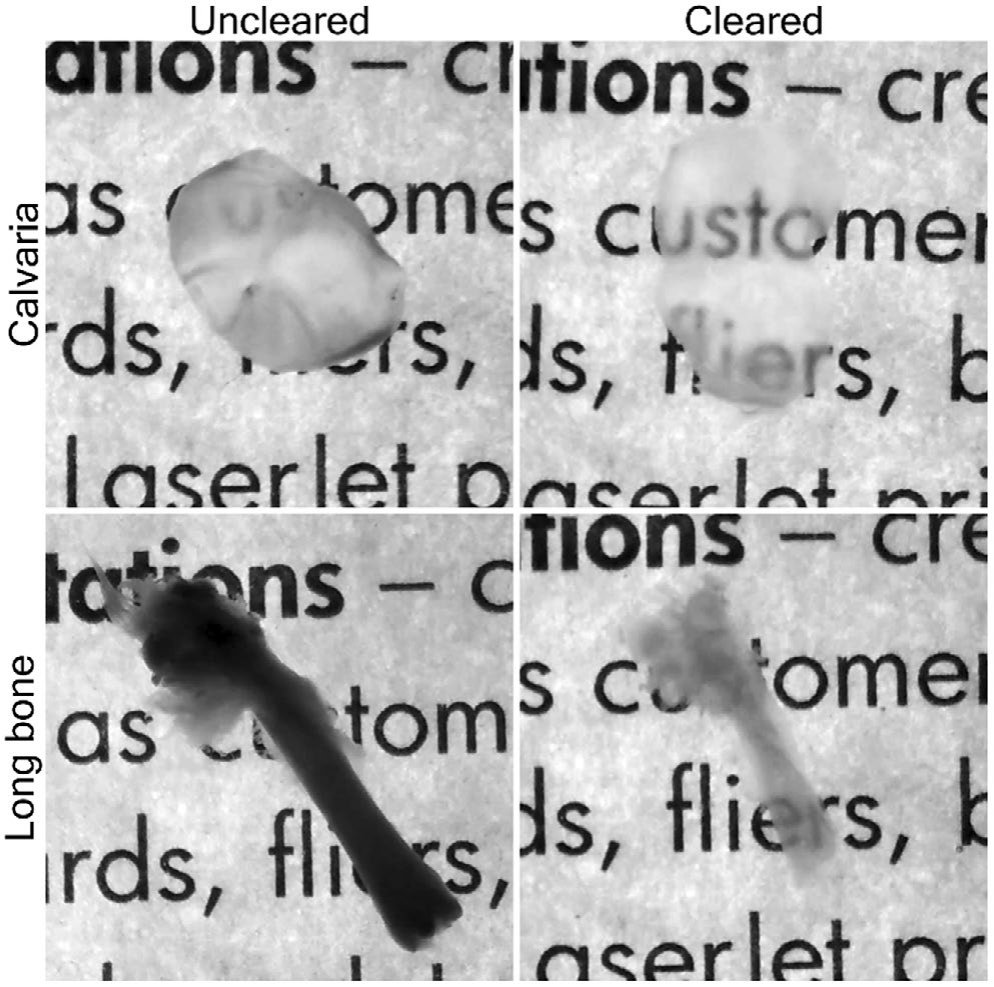
Representative images of murine calvaria (upper panels) and long bones with and without clearing PACT protocols.

Following SHG imaging, we carried out standard decalcification procedures for murine bone, using EDTA/formic acid to remove the calcified/mineral phase from these samples. We then processed the tissue through the Passive CLARITY (PACT) protocol, which involved impregnation of the tissue with monomer, followed by polymerisation, tissue clearing, fluorescent probe labelling and refractive index matching. Firstly, tissues were infiltrated with monomeric hydrogel solution A4P0, supplemented with 0.25% photoinitiator 2,20‐Azobis [2‐(2‐imidazolin‐2‐yl) propane] dihydrochloride for 4 days at room temperature. This monomer consists of acrylamide (0.5 mL), VA‐044 (12.5 mg), PBS 10× (0.5 mL) and dH_2_O (4 mL) and was chosen because it is relatively gentle (compared with other available reagents), non‐corrosive and is compatible with many fluorescent fluorophores and antibodies for immunohistochemistry. Samples were degassed with nitrogen for 5 min and incubated for 2–3 h at 37°C, in either a standard shaker or water bath, to initiate hydrogel polymerisation. Once the tissue‐hydrogel polymerisation was complete, tissues were then cleared by transferring samples to 50 mL tubes containing 8% SDS (sodium dodecyl sulphate) in 0.1 M of PBS (pH 7.5–8). The samples were left in this solution at 37°C until visually cleared (which took approximately between 3 and 7 days with the thin calvaria being cleared more quickly). This produced samples that were visibly transparent when compared with uncleared tissue (Figure 4). The actual processing time varied depending on sample thickness and composition and tissue type.

**FIGURE 5:**
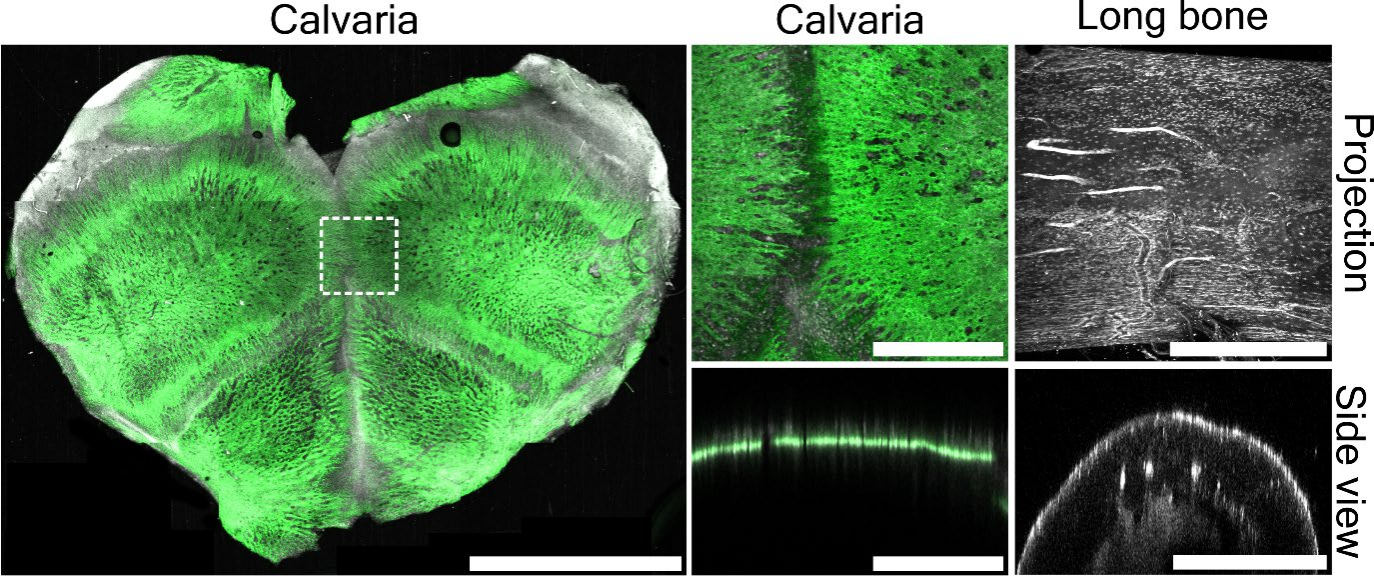
Representative images of rodent calvarium and long bone samples following clearing with PACT protocols, staining for osteoblast activity (AP, Green) and nuclei (DAPI, White) imaged with laser‐confocal microscopy. Left to right is an overview image (scale bar = 3 mm) with an inset showing the location of the Z projection and side view of the calvaria. On the right is a Z projection and side view of a long bone, nuclei only (DAPI, White). Side views are from the YZ orientation. All scale bars = 500 μm unless otherwise stated.

Once visually clear, the samples were then rinsed in a 0.1% PBS solution three times over 24 h. Then, where possible, fluorescent labelling was used to highlight specific cellular or extra‐cellular features of interest. For example, calvaria were probed for alkaline phosphatase (AP) which, in skeletal tissues, is a reliable marker for osteoblast activity and DAPI (4′,6‐diamidino‐2‐phenylindole) for nuclei. This fluorescent probe did not penetrate our long bone samples sufficiently well for reliable multi‐modal imaging and is thus omitted from this section of our report. Nuclear staining however was successful and reliable; thus, representative images of that are included in Figure 5.

Once calvaria samples were successfully stained for AP, refractive index matching was performed. Refractive index matching is an important step in the process, where the sample is immersed in a solution with the same refractive index as that of the tissue (1.48–1.49) (Treweek et al., 2015). When the refractive index of biological samples differs significantly from their mounting medium, imaging issues arise. Spherical aberrations, which degrade image clarity and accuracy, are a common consequence. These distortions are particularly pronounced in thick and intricate specimens, making deep tissue imaging challenging. Bone tissue has a high refractive index compared to other soft tissues in the body. Samples were placed in sRIMS (refractive imaging solution) overnight and replaced with fresh sRIMS and imaged between 2 and 4 days later. The sRIMS was composed of approximately 70% sorbitol solution in 0.02 M phosphate buffer with 0.01% sodium azide (pH adjusted to 7.5 with NaOH). A refractometer was used to monitor the refractive index of the solution and the concentration of sorbitol was adjusted as needed.

#### 2.2.4 Confocal and multiphoton microscopy

Once the tissue samples had been cleared, labelled and had undergone refractive index matching, they were mounted by creating a simple Blu Tack well on a cover slip containing the sample and adequate sRIMS. The well was then sealed using a second coverslip, to create a ‘sandwich’, this allowing easy imaging from both sides of the sample without remounting. This enabled proper fluorescent imaging using confocal microscopy and multiphoton imaging (both of which have the advantage of being possible to combine with SHG). Fluorescence labelling allows for high‐resolution image capture, an intact overview of the sample and as well as the use of multiple probes. Additionally, cell phenotypes and their interactions can be observed, and the 3D data has excellent resolution in the X, Y axes with course detail visible in the *z* axis. This is demonstrated in Figure 5 showing confocal imaging of ALP and/or nuclear staining to show cell phenotype. Briefly, a Carl Zeiss 710 NLO confocal microscope equipped with a W N‐Achroplan 10×/0.3 water immersion lens was used to image AP and DAPI. The sample was excited at 405 and 561 nm with emission collected in the ranges 410–585 and 585–735 nm for DAPI and AP, respectively. The Zen 2010 software was used for image collection with the overview images captured as a tiled image.

#### 2.2.5 Light sheet microscopy

Light sheet microscopy is a relatively recent imaging modality with excellent X, Y and Z resolution when multiple angles are collected and registered. The acquisition process for light sheet microscopy is comparatively fast when compared to point scanning confocal. However, processing the subsequent multi‐view light sheet data can be slow due to the large size and complexity (Gibbs et al., 2021). Here, images were acquired with a Light‐sheet Z.1 Microscope (Carl Zeiss, Germany) equipped with an EC Plan Neofluar 5×/0.16 objective lens with left and right illumination provided by two LSFM 5× NA 0.1 illumination objectives (Carl Zeiss, Germany). The sample was excited at 405 and 561 nm with emission separated using SP 490 and LP 505 nm filters. Using dual side illumination with pivot scan enabled, z stacks at four angles, 90° apart were captured with using dual PCO Edge 5.5 sCMOS cameras (PCO AG, Germany). A sheet thickness of 10.73um resulted in a spacing matrix of 1.23 × 1.23 × 5.03 μm in the X, Y and Z planes, respectively. The resulting dual‐side images were fused using the Zen 2014 SP1 Black‐Edition (Carl Zeiss, Germany) and the maximum fusion algorithm. Finally, multi‐view registration and fusion of the data set was performed in FIJI (Schindelin et al., 2012) using the Multiview Reconstruction (Preibisch et al., 2010) and BigStitcher plugins (Horl et al., 2019). The final resolution in XYZ after processing was isotropic, thus 1.23 μm in all directions.

#### 2.2.6 Bright‐field microscopy

The gold standard and clinically recognisable histological processing and staining of musculoskeletal tissues are well established in medical practice and research. Here we show that the cleared tissue used for confocal and light sheet microscopy yields very similar structures and details to the histological gold standard, but without the requirement for sectioning of the sample (Figure 7). As a final step in our process, the re‐embedding, sectioning and staining of cleared samples with safranin‐O was carried out to illustrate the utility of this approach. Images were acquired with a Nikon Eclipse 90i microscope equipped with a DS‐Ri1 camera and Plan Fluor 20× Objective in the Nikon NIS‐Elements Basic Research V3.10 software.

## 3 DISCUSSION

Here we describe a combination of laboratory‐based tissue preparation and imaging procedures/protocols that can enhance the existing suite of methods that are used to quantify musculoskeletal disease in bones/joints, such as osteoporosis and osteoarthritis in pre‐clinical models, or tissue‐engineered tissues/structures.

MicroCT is a well‐established and standardised method for determining bone properties and, in particular, bone tissue microstructural parameters (for both cortical and trabecular bone) in a variety of musculoskeletal disease models. This is an excellent and non‐destructive tool for assessing mineralised structures and features down to the micron scale. However, as a technology based on the simple principle of *x*‐ray attenuation (albeit coupled with advanced 3D reconstructive algorithms, etc.) microCT cannot easily provide information on biological processes or make use of biological labelling technologies (e.g. fluorescent probes) which are common in other areas of pre‐clinical imaging. Thus, combining this technology with others is a useful approach, but one which typically needs to be modified to bypass the difficulty of dealing with dense, often opaque, skeletal tissues.

Another laboratory‐based imaging modality that is useful for these tissues, in that it requires no staining or labelling, is second harmonic generation (SHG) imaging. Bone tissue is a composite material consisting of a fibre mesh of mostly collagen type I, integrated with hydroxyapatite mineral (Stock, 2015). While not directly mineralised, healthy cartilage tissue too depends on an intricate relationship between its ECM and a fibrillar mesh of collagen—in that case the collagen is of the type II variety. This relationship between fibrillar collagens and their surrounding ECM is disrupted in various musculoskeletal diseases such as osteoporosis, type II diabetes, Paget's disease and osteogenesis imperfecta. Therefore, techniques such as SHG that can reliably assess the organisation of the collagenous matrix microarchitecture are important. SHG is a label‐free non‐linear coherent imaging strategy applicable to non‐centrosymmetric structures like collagen fibres. The relative signal intensity in this method is dependent on collagen orientation relative to the excitation source. This method has been used before in similar applications but not, to our knowledge, in conjunction with other imaging modalities which were used and explored here.

We next explored the optimal method to introduce optical tissue clearing and imaging into our workflow. Skeletal tissue opaqueness derives from heterogeneous optical properties among its different components. Water has a refractive index (RI) of 1.33, while that of protein structures is higher (RI = 1.44) and that of the lipid family is slightly higher again (RI = 1.45) (Tainaka et al., 2014; Treweek & Gradinaru, 2016; Tuchin et al., 1997). These differing RI between different components scatter incoming light making imaging difficult, also different colouration (e.g. between bone, marrow and cartilage) also add to the difficulty. Calcified tissue, namely hydroxyapatite, and collagen further block light transmission in bone. There are many tissue‐clearing techniques now that are able to achieve tissue transparency (including 3DISCO, FluoClear, uDISCO, CLARITY, CUBIC, PACT, SWITCH, CUBIC‐R and Bone CLARITY) (Chen et al., 2015, Chung et al., 2013, Chung & Deisseroth, 2013, Dodt et al., 2007, Erturk et al., 2012, Greenbaum et al., 2017, Hama et al., 2011, Hama et al., 2015, Ke et al., 2013, Kubota et al., 2017, Murray et al., 2015, Pan et al., 2016, Schwarz et al., 2015, Treweek et al., 2015), but most operate through the same set of physical principles. Essentially transparency can be achieved by eliminating RI mismatch and decolourising heavily pigmented elements of tissue. In general, tissue‐clearing methods can be classified into either organic solvent‐based or aqueous reagent‐based methods. Organic solvent‐based approaches obtain high tissue transparency by using clearing medium with high RI (RI >1.50). Most of the aqueous reagent‐based methods have lower RIs (RIs <1.49). Aqueous reagent‐based clearing methods including CLARITY were developed for clearing brain tissue. Initially Bone CLARITY was successfully able to clear long rodent bones, but the process was long and time‐consuming and reagents were costly (Greenbaum et al., 2017). The subsequent iteration was a passive clearing technique (PACT) that could achieve optical transparency with limited equipment and hands‐on processing time and without a requirement for electrophoretic tissue clearing. The early CLARITY methods significantly advanced our ability to determine and quantify the three‐dimensional relationships between biological structures. However, its relatively harsh treatments are not amenable to clearing rodent bones. For this reason, various methodologies have been developed specifically for mouse bone tissue. For instance, Bone‐CLARITY (an optimised version of PACT‐deCAL) requires roughly 4 weeks to achieve optical transparency. The PACT protocol that we used involves a hybrid tissue hydrogel embedded with 4% acrylamide and photoinitiator (0.25% VA‐044). The transparent tissue is generated with a final additional incubation in 8% sodium dodecyl sulphate (SDS) clearing solution after embedding. The tissue damage and expansion that occurs during clearing of the hard bone tissue with these methods can be problematic but was minimal in this work.

**FIGURE 6:**
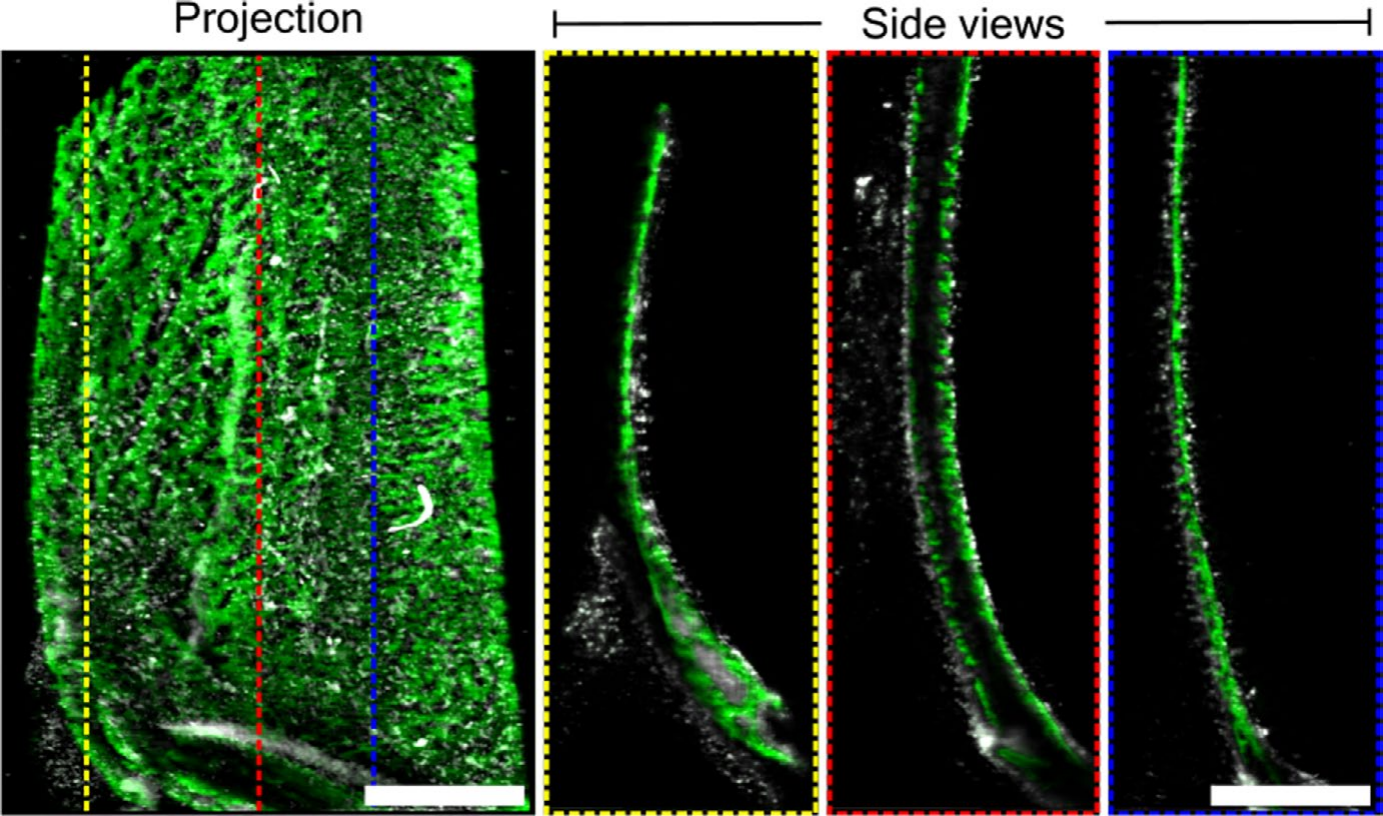
Representative light sheet microscopy images of rodent calvarium following clearing protocols, staining for osteoblast activity and nuclei (AP, green | DAPI, white). Side views are from the YZ orientation with the approximate position indicated on the projection image using colour‐coded lines. Scale bar = 500 μm.

**FIGURE 7:**
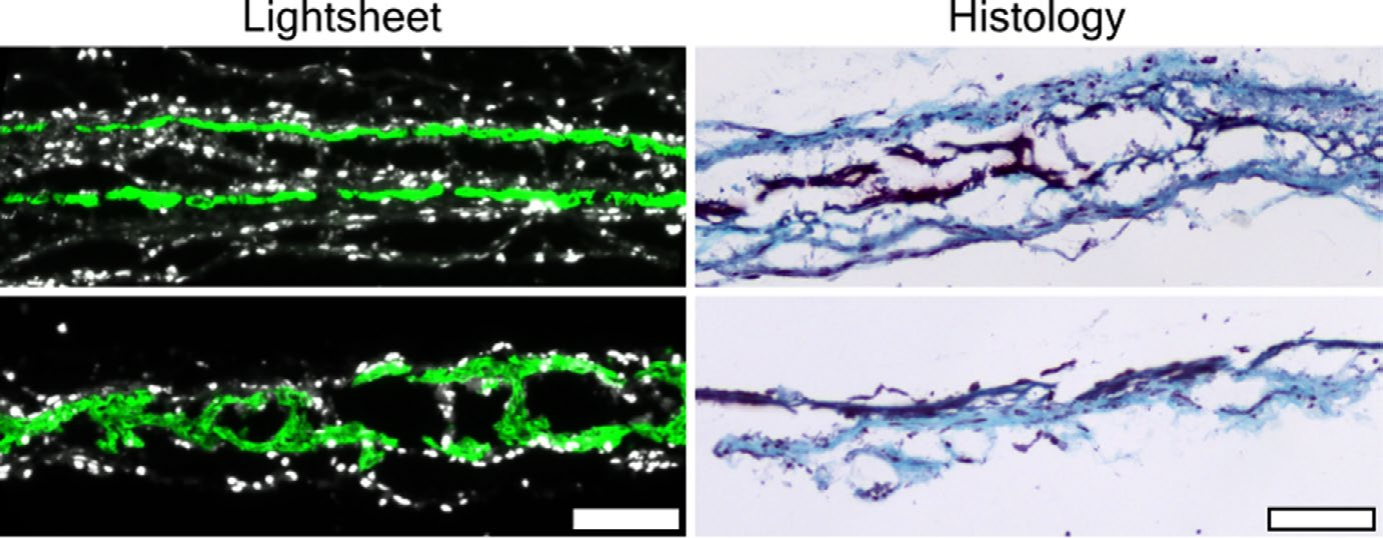
Comparative images taken in sagittal section through the calvarium showing the resolution and detail that can be obtained using light sheet microscopy (left panels: AP, green | DAPI, White) is strikingly similar to that obtained using standard histological processing of the same tissue sections (e.g. sagittal sections through the calvarium) (right panels: standard paraffin‐embedded sections, safranin‐O staining) while maintaining the structural integrity of the tissue, and avoiding the need for sectioning. Scale bar = 100 μm.

As our initial proof‐of‐concept studies demonstrated, we were able to reliably stain for, and image, alkaline phosphatase in our samples, which is a standard marker for osteoblast activity, in our calvarial and long‐bone samples. As expected the staining procedures worked well in the calvarium, since this is a thin and more easily cleared tissue. However, the fluorescent label did not penetrate as well in the long bone samples. Nuclear labelling however was equally successful in both and can be seen in Figure 5, which was captured using laser confocal microscopy. However, for this reason, our last round of staining and imaging with light sheet microscopy (LSM) was carried out only on calvarium samples. Figure 6 shows calvarium, stained for AP activity viewed from a variety of angles and showing excellent resolution in all 3 axes. Furthermore, in these samples we also subsequently processed the tissue for standard histological staining and microscopy as a comparison. Figure 7 shows excellent comparison between the standard stained histological sections, and the images taken using PACT and LSM. The latter of which can be achieved with relatively straightforward clearing steps, and completely bypassing the time‐consuming and technically challenging embedding, sectioning and staining procedures.

In conclusion, our proposed work‐flow and protocol significantly improves the options available for processing and imaging of hard/mineralised tissues of the musculoskeletal system for specific applications. Furthermore, other tissues of the musculoskeletal system are also amenable for processing and imaging in a similar way. Arguably, the primary focus of our application in this report (e.g. mineralised bone tissue) is the most difficult to prepare and process for these techniques (with the possible exception of dental/tooth/enamel tissues). Thus modifying these methods for other tissues should be very feasible, with some minor optimisation steps likely being required. We propose a more efficient system than traditional passive clearing methods, which requires minimal equipment and hands‐on processing time. Our work‐flow suggests that this is a feasible and useful addition to the landscape of imaging in musculoskeletal disease research.

## AUTHOR CONTRIBUTIONS

**Anya König:** Data collation, manuscript concept/drafting and critical revision. **Brenton Cavanagh:** Concept design, data acquisition, data analysis and interpretation, critical revision of the manuscript, drafting of the manuscript. **Isabel Amado:** Data acquisition, technical development, technical/student supervision, data analysis and interpretation. **Bohnejie Ogon:** Technical preparation and data acquisition. Amit Kalra: Technical preparation and data acquisition. **Paige V. Hinton:** Data acquisition, data analysis. **Oran D. Kennedy:** Concept design, data acquisition, data analysis and interpretation, critical revision of the manuscript, drafting of the manuscript.

## DATA AVAILABLITY STATEMENT

Imaging data associated with this manuscript is made available as per the Institutional Data‐Sharing and Open‐Access guidelines of the RCSI University of Medicine and Health Sciences.
